# The Important and Untapped Role of the Surgeon in Cardiac Rehabilitation

**DOI:** 10.1016/j.atssr.2025.02.001

**Published:** 2025-02-28

**Authors:** Usman Khan, Steven J. Keteyian, Francis D. Pagani, Michael P. Thompson

**Affiliations:** 1 Department of Cardiac Surgery, Michigan Medicine, Ann Arbor, Michigan; 2 Division of Cardiovascular Medicine, Henry Ford Health, Detroit, Michigan; 3 Center for Healthcare Outcomes and Policy, University of Michigan, Ann Arbor, Michigan

Cardiac rehabilitation (CR) is a comprehensive, medically supervised program that affords clinical benefits for cardiac surgical patients, including those undergoing coronary artery bypass grafting, heart valve procedures, and heart transplantation.^1^^,^^2^ Although CR typically occurs in 3 phases—inpatient, early outpatient, and maintenance, the outpatient phase focuses on clinical guidelines for qualifying patients that recommend a total of 36 sessions over a 3- to 4-month period. Outpatient CR, primarily thought of as monitored exercise training, is a multidisciplinary therapy that includes education on heart-healthy living, medication education, and counseling to manage stress and enhance mental health to aid in recovery and reduce the risk of secondary cardiovascular events. Evidence from meta-analyses and observational data shows that participation in cardiac rehabilitation not only enhances exercise capacity but also significantly reduces mortality, with studies suggesting a 40% reduction in 2-year mortality after coronary artery bypass grafting, along with improvements in both functional capacity and quality of life for patients who undergo heart valve procedures and for transplant recipients.^1^, ^2^, ^3^ Additionally, CR participation has been linked to reductions in hospital readmissions and lower overall health care costs, with some studies suggesting up to a 25% reduction in hospital readmissions among CR participants.^4^^,^^5^ Delays in participation mean that patients miss out on these well-documented benefits, thereby prolonging their recovery, increasing their risk for secondary events, and potentially leading to worse long-term outcomes.

Despite the documented benefits and guideline recommendation for CR, participation in this therapy after cardiac surgery remains significantly underused. About one-half of cardiac surgical patients never attend a CR facility, and only 10% to 30% complete all 36 recommended sessions that are shown to provide the largest clinical benefit.^3^^,^^4^ There is extraordinary variation in CR enrollment across hospitals after cardiac surgery, even after accounting for patient demographics and clinical factors.^5^ These findings suggest that various practices, beliefs, and systems may be in place that affect CR enrollment and attendance. Furthermore, relative to other CR-qualifying conditions, cardiac surgical patients typically have a delay of several weeks before beginning CR as a result of recovery and healing from the surgical procedure.^4^ Finally, well-documented disparities in CR use across vulnerable populations, such as women, minorities, and socioeconomically disadvantaged patients, may exacerbate long-standing disparities in cardiac surgical outcomes.^4^ These disparities highlight the need for a more consistent and equitable approach to CR referral and enrollment to ensure that all patients benefit from this crucial aspect of postsurgical care.

Although most patients start CR once they are beyond the surgeon’s care, the surgeon can and should play an important and collaborative role in addressing common barriers to CR participation that start while the patient is still in their care (Table). First, surgeons could support the implementation of care processes that mitigate referral and CR enrollment delays. Delays in referral to CR for cardiac surgical patients lead to longer delays in CR initiation and lower overall participation.^6^ In some cases, referrals to CR are not placed until postoperative follow-up appointments, and this can add to the time between scheduling the first visit and participation in CR. Strategies to address delays in wait times for referral and initiation include automatic referrals and CR liaison programs. Automatic referrals can be easily implemented into electronic medical record order sets and have been shown to improve overall participation rates. Liaison programs can also bridge the gaps among the patient, the surgical team, and CR staff by educating patients about CR, discussing potential barriers to enrollment, and ensuring referral and scheduling of their first session.^7^ Another key advocacy point is prehabilitation. These structured interventions can be designed to optimize patients before surgery, an approach that has gained popularity in general surgery but remains understudied in cardiac surgery. Because CR already focuses on exercise, education, and risk factor modification, incorporating a structured prehabilitation approach before cardiac surgery could enhance recovery outcomes. Although data are currently limited, future research should explore whether a preoperative introduction to CR could improve postsurgical participation rates and recovery trajectories.

**Table:** Opportunities for Surgeon Engagement in Supporting Enrollment in Cardiac Rehabilitation

Mechanism of Action	Specific Goals	Opportunities for Surgeon Engagement
Supporting care process implementation	Mitigating delays and gaps in enrollment	Endorse the use of automatic referrals to CR for cardiac surgical patients.
		Support the use of CR liaison programs.
Strong endorsement	Motivating and educating patients	Discuss the benefits of CR with patients during preoperative, perioperative, and postoperative follow-up visits.
		Directly address patients’ concerns and questions about the CR process.
Advocating for CR programs	Supporting CR programs within the health system	Ensure that CR programs have sufficient staff and resources.
		Support the development of institutional funds for patients with financial needs.
		Discuss and advocate for the clinical and business case for CR with health system administration.

CR, cardiac rehabilitation.

Surgeons can also significantly affect CR participation by strongly endorsing it during preoperative, perioperative, and follow-up visits. Awareness of the benefits of CR and a strong endorsement by the physician have been shown to contribute positively toward downstream CR referral and participation.^8^ Surgeons can address this gap by explaining how CR can improve recovery outcomes, enhance exercise tolerance, and reduce future heart problems by ensuring the success of the surgery they just performed. For elective surgical patients, discussing CR as part of the recovery pathway may further reinforce expectations about CR participation for patients. Through these discussions, surgeons can help address any worries or misunderstandings patients may have about CR, such as fears about engaging in exercise shortly after the procedure or doubts about its effectiveness. Caring for cardiovascular patients is inherently team based, with surgeons playing a crucial role in the perioperative phase. However, surgeons’ involvement should not end there. By remaining engaged beyond the confines of the perioperative phase, surgeons can help facilitate the transition into CR and ensure continuity of care. Their endorsement and direct involvement in discussions about CR can lead to increased patient engagement and improved recovery outcomes. Engaging surgeons in ongoing CR discussions can foster interdisciplinary collaboration among cardiac surgeons, cardiologists, and rehabilitation specialists, ultimately benefiting patient outcomes.

Finally, advocating within the health system for the necessary resources to support CR programs is essential. This means supporting efforts to secure patient funds, expanding CR facilities and capacity, and investing in CR staff. The COVID-19 pandemic led to hundreds of CR program closures nationwide, and participation rates have not yet fully recovered.^9^ In an interview with staff of a hospital with high rates of CR participation, strong buy-in from physician and administrative leaders was cited as a critical component to their success.^8^ As a primary source of CR referrals, surgeons have an important voice that can be used to advocate for the resources needed to support CR programs. The surgeon’s voice can help highlight CR’s clinical and business case as cost-effective therapy for their patients and health systems.^10^ Supporting these efforts can make a big difference in increasing CR enrollment and ensuring that all patients benefit from this essential part of their recovery.

There is an imminent need to improve participation in CR nationally to ensure the surgical success and recovery of patients undergoing cardiac surgery. Active involvement in improving CR participation improves patient recovery and outcomes and increases the effectiveness and efficiency of the procedures provided to the patients. In partnership with the broader cardiovascular care teams, surgeons play a crucial role in helping patients benefit from CR and improving participation rates. Surgeons can significantly increase CR participation by setting up supportive care processes for early enrollment, providing strong endorsement for CR, and advocating for needed resources within health systems.

## References

[1] Sibilitz K.L., Berg S.K., Tang L.H. (2016). Exercise-based cardiac rehabilitation for adults after heart valve surgery. Cochrane Database Syst Rev.

[2] Heran B.S., Chen J.M., Ebrahim S. (2011). Exercise-based cardiac rehabilitation for coronary heart disease. Cochrane Database Syst Rev.

[3] Bauer T.M., Yaser J.M., Daramola T. (2023). Cardiac rehabilitation reduces 2-year mortality after coronary artery bypass grafting. Ann Thorac Surg.

[4] Keteyian S.J., Jackson S.L., Chang A. (2022). Tracking cardiac rehabilitation utilization in Medicare beneficiaries: 2017 update. J Cardiopulm Rehabil Prev.

[5] Fliegner M.A., Hou H., Bauer T.M. (2025). Interhospital variability in cardiac rehabilitation use after cardiac surgery among Medicare beneficiaries. J Thorac Cardiovasc Surg.

[6] Marzolini S., Blanchard C., Alter D.A., Grace S.L., Oh P.I. (2015). Delays in referral and enrolment are associated with mitigated benefits of cardiac rehabilitation after coronary artery bypass surgery. Circ Cardiovasc Qual Outcomes.

[7] (2024). Cardiac rehabilitation change package. 2nd ed. Centers for Disease Control and Prevention. https://millionhearts.hhs.gov/tools-protocols/action-guides/cardiac-change-package/index.html.

[8] Thompson M.P., Yaser J.M., Forrest A., Keteyian S.J., Sukul D. (2022). Evaluating the feasibility of a statewide collaboration to improve cardiac rehabilitation participation: the Michigan Cardiac Rehab Network. J Cardiopulm Rehabil Prev.

[9] Varghese M.S., Beatty A.L., Song Y. (2022). Cardiac rehabilitation and the COVID-19 pandemic: persistent declines in cardiac rehabilitation participation and access among US Medicare beneficiaries. Circ Cardiovasc Qual Outcomes.

[10] Edwards K., Jones N., Newton J. (2017). The cost-effectiveness of exercise-based cardiac rehabilitation: a systematic review of the characteristics and methodological quality of published literature. Health Econ Rev.

